# Bullous Pemphigoid and Human Leukocyte Antigen (HLA)-DQA1: A Systematic Review

**DOI:** 10.7759/cureus.39923

**Published:** 2023-06-03

**Authors:** Roksana Hesari, Dylan Thibaut, Nina Schur, Shivani Thoutireddy, Ryan Witcher, Elyse Julian

**Affiliations:** 1 Osteopathic Medicine, Lake Erie College of Osteopathic Medicine, Bradenton, USA; 2 Dermatology, Leading Edge Dermatology, Plantation, USA

**Keywords:** allele, meta-analysis, systematic review, hla-dqa1, dqa1, bullous pemphigoid, human leukocyte antigen, mhc class ii, mhc, hla

## Abstract

Bullous pemphigoid (BP) is an autoimmune blistering disease that mainly affects the elderly. The human leukocyte antigen (HLA) system is believed to be one of the genetic factors involved in the development of BP. The connection between major histocompatibility complex class II, specifically HLA-DQA1, and BP remains inconclusive. The objective of this review is to find potential associations between BP and HLA-DQA1 alleles, identify the HLA-DQA1 alleles associated with an increased or decreased risk of developing BP, and highlight literature gaps for future research. The Preferred Reporting Items for Systematic Reviews and Meta-Analysis (PRISMA) guidelines were used to conduct a literature review. Databases used included PubMed/MEDLINE, Google Scholar, Embase, and Cochrane Library. Only studies written in English and conducted after 2000 that investigated the association between HLA-DQA1 and BP in human subjects were included. Odds ratios were calculated from the data provided in the studies, and a meta-analysis was conducted using Review Manager (The Cochrane Collaboration, London, United Kingdom) and MetaXL (EpiGear International Pty Ltd., Queensland, Australia) software. The systematic review found five eligible studies, and all were included in the meta-analysis. Results show an increased odds for BP in the HLA-DQA1*05:05 loci (odds ratio (OR) = 2.25; 95% confidence interval (CI) = 1.80, 2.80) and decreased odds for BP in the HLA-DQA1*02:01 loci (OR = 0.50; 95% CI = 0.36, 0.70). Further research is needed to confirm these findings and explore the potential clinical implications for personalized medicine approaches in BP patients.

## Introduction and background

Bullous pemphigoid (BP) is an autoimmune blistering disease that has rising incidence [1]. This dermatologic condition is identified by the presence of generalized urticarial plaques and tense subepidermal blisters [2]. Despite its overall rarity, BP stands out as the most common among autoimmune bullous skin disorders [3]. Of particular concern is the fact that it primarily affects the elderly, as the aging population is rising globally [2,4].

The hallmark of BP is the formation of autoantibodies against components of the basement membrane zone of the skin, such as BP180 and BP230 [3,4]. The recognition of these epitopes by T-cells induces the production of cytokines, and subsequently, B cells are stimulated to produce autoantibodies. The binding of these autoantibodies to the basement membrane zone leads to complement activation and the subsequent recruitment of inflammatory cells, resulting in tissue damage and blister formation [4,5]. The diagnosis of BP relies on histopathologic analysis from the edge of a blister and direct immunofluorescence demonstrating a linear pattern at the dermal-epidermal junction [2].

The pathogenesis of BP is complex, and the disease is believed to result from the interplay of various predisposing and precipitating factors, including genetics, comorbidities, and aging [2]. Among these factors, one that has been extensively studied in the context of autoimmune disorders like BP is the human leukocyte antigen (HLA) system [5]. HLA is a complex system of genes located on chromosome 6 that encode for proteins involved in the regulation of the immune system [6,7]. Genetic variation in HLA and its major histocompatibility complex (MHC) genes has been linked to a wide spectrum of immunological diseases [6-8]. In fact, MHC is recognized as the region of the genome associated with the greatest number of human diseases [8].

Recent developments in next-generation sequencing and omics technologies have made autoimmune bullous diseases like BP the focus of genetic and high-throughput data studies [5]. Due to this, population studies have found that MHC class II alleles are associated with BP in various ethnic populations, including British, German, Japanese, Chinese, and Iranians [2]. In particular, MHC class II genes, such as HLA-DR and HLA-DQ, have been shown to be prevalent in patients with BP [5]. Notably, there is growing evidence suggesting that specific HLA-DQA1 alleles are associated with an increased risk of developing BP. However, the evidence remains inconclusive, and a meta-analysis is needed to synthesize the available literature and provide a comprehensive overview of the association between HLA-DQA1 and BP. The specific objectives of this review are to systematically review the literature on the association between BP and HLA-DQA1 alleles, to identify the HLA-DQA1 alleles that are associated with an increased or decreased odds of developing BP, and to identify gaps in the current literature and highlight areas for future research.

By providing a systematic review of the available literature on the association between HLA-DQA1 and BP, this paper aims to improve our understanding of the genetic factors involved in the development of BP and ultimately facilitate the identification of carriers of the condition. This review will also contribute to the identification of potential triggers that may exacerbate or induce BP in individuals with a predisposing genetic profile. Overall, the results of this systematic review have the potential to inform clinical practice and contribute to the development of personalized medicine approaches for patients with BP.

## Review

Methodology 

The Preferred Reporting Items for Systematic Reviews and Meta-Analysis (PRISMA) guidelines were used to conduct the literature review [9]. To meet the eligibility requirements for this review, the study had to be written in English, conducted after the year 2000, and include studies that investigated the association between HLA-DQA1 and BP in human subjects and that were accessible through open access or institutional access. Studies that included multiple measurements of different populations and meta-analyses or those that could not be accessed were excluded. Databases used for this study include PubMed/MEDLINE, Google Scholar, Embase, and Cochrane Library. Institutional access will be used for journals that may not be open access. The exact search terms used included “bullous pemphigoid” AND “HLA” AND “DQA1.”

Three independent researchers conducted the selection process, and the data were compiled in a shared Excel document. Any questions related to the authenticity or accuracy of the data were referred to the other researchers involved in the study. The principal investigator will be consulted for the final decision if a consensus cannot be reached.

**Figure 1:**
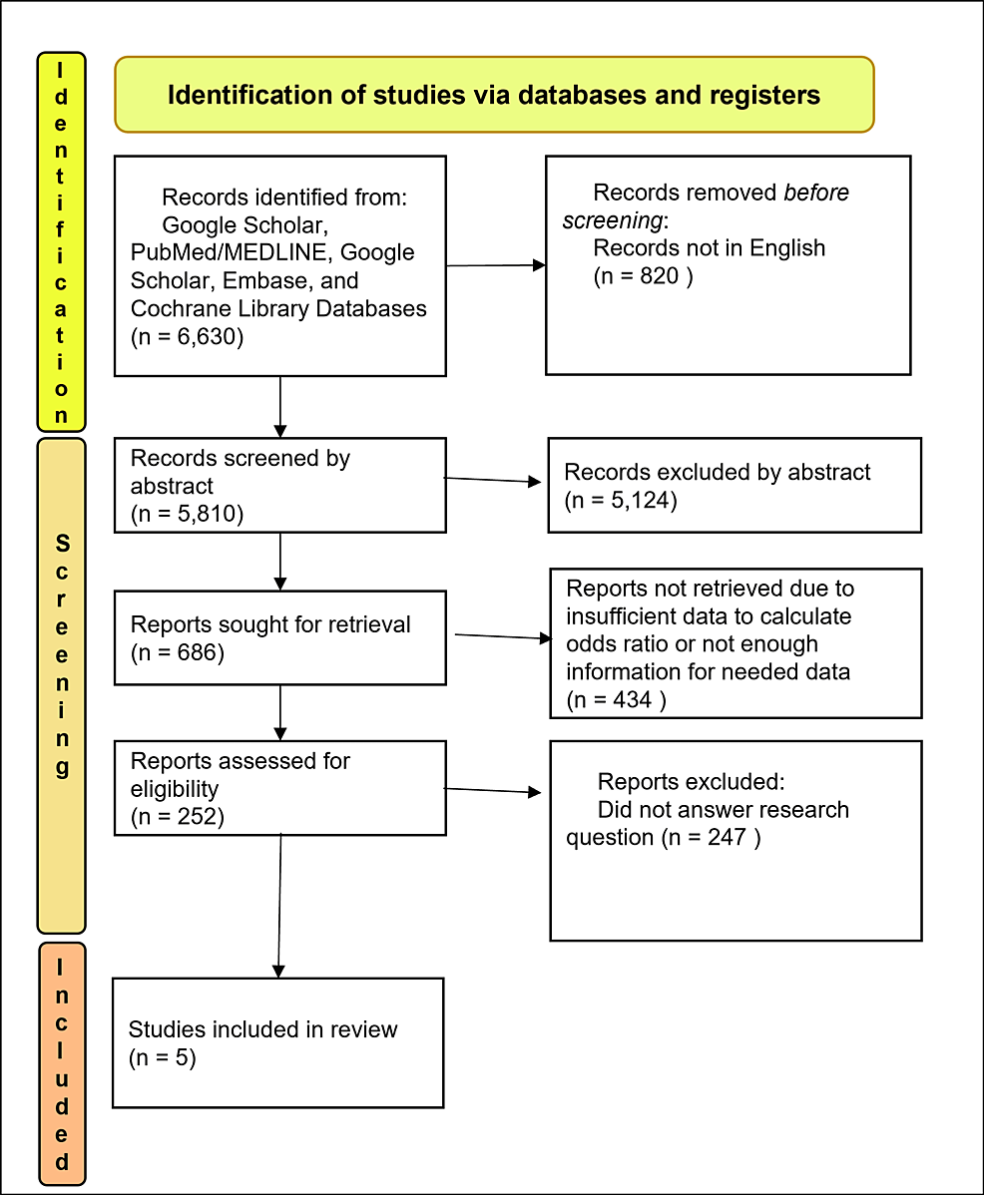
PRISMA flow diagram PRISMA:  Preferred Reporting Items for Systematic Reviews and Meta-Analysis

The results of this meta-analysis were calculated based on the data items collected from each selected study, including the number of cases of BP, the number of cases with BP and HLA-DQA1 alleles, the number of controls and the number of controls with the HLA-DQA1 alleles, and calculable or provided odds ratio (OR) with associated confidence intervals (CIs) of each individual allele. The database search results and evaluation for inclusion criteria are shown in Figure 1. 

Only studies that reported ORs or provided enough data to calculate the OR were considered eligible and were included in the meta-analysis. Data were collected and compiled into tables for each HLA-DQA1 allele studied. Review Manager 5.4 (RevMan Version 5.4, The Cochrane Collaboration, Released 2020, London, United Kingdom) was used for sample analysis based on the sample size [10]. A p-value below 0.05 provided enough evidence of a difference between the odds. I² was used to evaluate heterogeneity; a value below or equal to 50% was considered evidence of low heterogeneity [11]. MetaXL (EpiGear International Pty Ltd., Queensland, Australia) was used for analysis based purely on the OR with associated CIs. The ORs obtained from the forest plot analysis and a 95% CI were used to measure the outcome of this study. The National Institutes of Health (NIH) quality assessment tool of case-control studies was used to assess the risk of bias in individual studies [12]. A Doi plot and Luis Furuya-Kanamori (LFK) index were generated using MetaXL as a quantitative measure of bias [11]. A protocol was created and followed prior to starting the analysis [13].

Results

The present meta-analysis found five studies that met the selection criteria and compared a total of 707 BP patients with 2500 controls [14-18]. The studies were published between 2013 and 2021. The analysis focused on two alleles, namely, HLA-DQA1*02:01 and HLA-DQA1*05:05, and a combined OR and 95% CI were created for each of the HLA alleles using the inverse variance (IV) random effect model.

To evaluate the internal validity of each study, the NIH quality assessment tool for case-control studies was utilized. The responses for each study are presented in Table 1, arranged according to the corresponding question number in the quality assessment tool. Out of the five studies analyzed in the meta-analysis, two demonstrated good internal validity, whereas three exhibited fair internal validity. The studies with fair internal validity lacked justification for their sample sizes, did not include concurrent controls, or failed to select control samples from the same populations.

**Table 1:** Bias assessment and study data NIH Bias Assessment Tool was used [12] NR: Not Recorded, Y: Yes, N: No, NIH: National Institutes of Health 1. Research question with the goal 2. Defining study populations clearly 3. Sample size justification 4. Groups recruited from the same population 5. Specify and apply inclusion and exclusion criteria 6. Defined description of cases and controls 7. Random sampling 8. Concurrent controls used 9. Exposure assessed prior to the outcome measurement 10. Details of exposure measures 11. Blindness of exposure assessors 12. Adjustment for confounding variables

Study	Case total (N)	Control total (N)	Sample	Bias	1	2	3	4	5	6	7	8	9	10	11	12
Chagury et al., 2018 [14]	34	594	Brazilian	fair	Y	Y	NR	N	Y	Y	Y	N	NR	Y	NR	Y
Fang et al., 2017 [15]	105	420	Chinese Hans	good	Y	Y	NR	Y	Y	Y	Y	Y	NR	Y	NR	Y
Esmaili et al., 2013 [16]	50	180	Iranian	fair	Y	Y	NR	N	Y	Y	Y	Y	NR	Y	NR	Y
Schwarm et al., 2021 [17]	446	433	German	fair	Y	Y	NR	Y	Y	Y	NR	N	NR	Y	NR	NR
Ujie et al., 2018 [18]	72	873	Japanese	good	Y	Y	Y	Y	Y	Y	Y	NR	Y	Y	NR	Y

**Figure 2:**
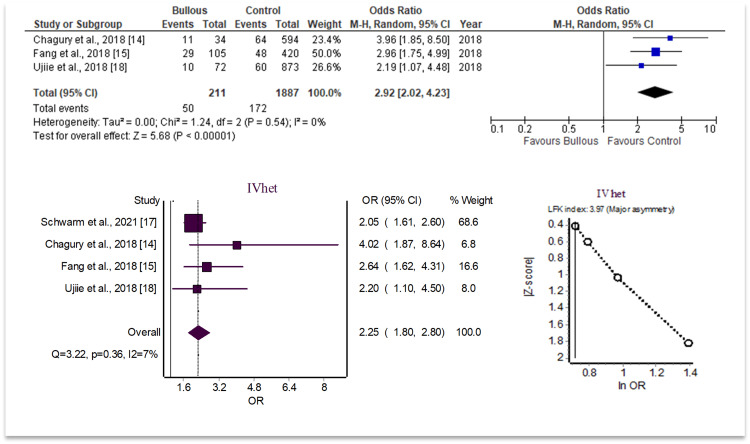
HLA-DQA1*05:05 analysis data

As there were two separate analyses conducted for each HLA allele, one including studies without sample size data versus a secondary analysis using ORs and confidence internal analysis alone, each allele will be analyzed separately with both findings indicated together. The findings of the meta-analysis using the sample size data revealed that the odds of BP were increased in patients with HLA-DQA1*05:05 (OR = 2.92; 95% CI 2.02, 4.23; I^2^ = 0%; p<0.05). Secondary analysis including OR-specific data found similar results with more included studies. When including these studies in a separate analysis, the odds of BP were increased in patients with HLA-DQA1*05:05 (OR = 2.25; 95% CI 1.80, 2.80; I^2^ = 7%; p<0.05). These results are summarized in Figure 2.

**Figure 3:**
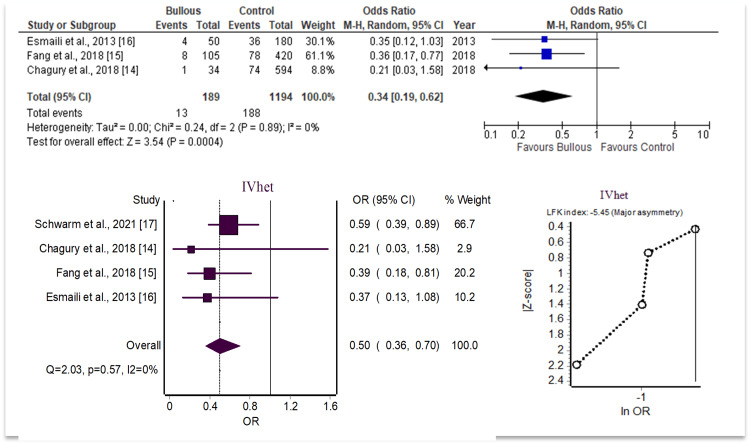
HLA-DQA1*02:01 analysis data

On the other hand, the odds of BP were conversely decreased in patients with HLA-DQA1*02:01 when using sample size comparison with a forest plot (OR = 0.34, 95% CI [0.19, 0.62], I^2^ = 0%, p<0.05). When only comparing OR and CI through a secondary analysis with additional studies included, the odds of BP were decreased in patients with HLA-DQA1*02:01 (OR = 0.50, 95% CI [0.36, 0.70], I^2^ = 0%, p<0.05). These results are summarized in Figure 3.

To evaluate the potential for publication bias or systematic heterogeneity, the LFK index and Doi plot were utilized. The LFK index is a useful technique for quantifying the asymmetries between individual studies analyzed on one Doi plot. Values above 2 or below -2 indicate major asymmetry and strongly suggest publication bias. Values between 1 to 2 and -1 to -2 represent minor asymmetry and a mild possibility for publication bias. The LFK indices for the two significant alleles showed major asymmetry, with the review of the HLA-DQA1*05:05 found to have an LFK of 3.97 and the review of HLA-DQA1*02:01 found to have an LFK of -5.45.

Discussion

In previous studies, there has been evidence of genetic linkage between HLA-DQA1 genes and increased susceptibility to developing BP. This meta-analysis focused on determining which of the HLA-DQA1 alleles provided the greatest linkage in BP susceptibility. Results showed an increased OR for HLA-DQA1*05:05 loci (OR = 2.25; 95% CI = 1.80, 2.80) and decreased OR for HLA-DQA1*02:01 loci (OR = 0.50; 95% CI = 0.36, 0.70). Both were statistically significant (p<0.05) with low heterogeneity. This suggests that HLA-DQA1*05:05 is associated with increased susceptibility to BP, whereas HLA-DQA1*02:01 is associated with protection against BP.

BP is a cutaneous autoimmune disorder that results in a 3.6-fold higher risk of death when compared to individuals without the disease of the same age and biological sex [19]. As noted in the introduction, many non-modifiable patient-specific factors have been associated with the development of BP, such as the presence of comorbid conditions and genetics [2,20]. Specifically, patients diagnosed with Parkinson's disease are three times more likely to have BP, and patients with multiple sclerosis are more than 12 times more likely to have BP [21]. Alternatively, there are modifiable triggers that are known to also trigger the development of BP, such as exposure to certain drugs. For example, BP has been reported as an adverse effect following the prescription use of both dipeptidyl peptidase IV inhibitors and programmed cell death-1/programmed death ligand-1 checkpoint inhibitors [22,23].

One potential advantage of demonstrating a link between certain HLA subtypes and BP is that physicians can utilize genetic testing as a diagnostic tool to preemptively identify individuals with a predisposing genetic profile to developing BP. Identifying susceptible patients early can optimize the personalization of medical treatment plans and result in avoiding exposure to potential triggers whenever possible. Emerging research suggests that specific HLA haplotypes, notably DRB1*08 and DQB1*06, may exhibit protective effects against the development of BP [24]. This discovery holds significant promise, particularly for individuals who have relatives affected by BP and are apprehensive about their vulnerability to the development of BP when exposed to the same triggering factors. Other advantages to identifying individuals with an increased HLA susceptibility to BP may include the establishment of potential susceptibilities to the later development of comorbid conditions. For example, a recent study of patients with multiple sclerosis showed a correlation between the presence of the HLA-DQA1*04:01 allele and greater lesion load on T2 MRI imaging, which suggests a higher risk of disease severity in individuals with the allele [25]. 

The limitations of the studies analyzed in this meta-analysis should be considered when interpreting the statistical data. Although meta-analysis findings showed significance and low heterogeneity, a major asymmetry was found in the LFK index on Doi plots. This suggests potential bias and is a limitation. To mitigate this, an additional method of bias assessment was utilized, and a sensitivity analysis was performed. Only five studies met the inclusion criteria, so there were low overall sample sizes for analysis. Additional database usage specific to this topic would likely help find more studies for inclusion or may have provided additional context to findings. The search for articles was also limited to articles published in the English language, removing potential studies for inclusion.

Despite these limitations, the findings of the meta-analysis suggest that HLA-DQA1*05:05 is a genetic factor that increases susceptibility to BP. This research may be clinically significant in identifying individuals at risk of developing BP based on their HLA profile and tailoring personalized treatment approaches. However, further research, including larger studies with more representative samples, may be needed to confirm these results. It may also be important to investigate potential biological mechanisms underlying the association, as this could provide insights into the development and progression of the disease.

## Conclusions

The goal of this meta-analysis is to assess the strength of HLA-DQA1 allele associations with BP. Our findings suggest that the HLA-DQA1*05:05 allele is associated with increased odds of developing BP, whereas the HLA-DQA1*02:01 allele is associated with decreased odds of developing BP. Future research should evaluate larger population sizes to confirm this linkage. In addition, future research should examine the potential utility of HLA genetic screening as a method of identifying BP susceptibility, thereby providing avenues for individualized treatment to prevent triggers resulting in the disease.
